# Inhibitory Effect of Sulfated Polysaccharide from *Codium edule* P.C. Silva Against 2,4-Dinitrofluorobenzene (DNFB)- Induced Allergic Contact Dermatitis on Female BALB/c Mice

**DOI:** 10.34172/apb.2022.042

**Published:** 2021-02-06

**Authors:** Martin Raemond Brondial Mallabo, Mary Jho - Anne T. Corpuz, Reginald B. Salonga, Ross D. Vasquez

**Affiliations:** ^1^The Graduate School, University of Santo Tomas, Manila, Philippines.; ^2^Senior High School, University of Santo Tomas, Manila, Philippines.; ^3^Department of Pharmacy,Faculty of Pharmacy, University of Santo Tomas, Manila, Philippines.; ^4^Research Center for Natural and Applied Sciences, University of Santo Tomas, Manila, Philippines.; ^5^Institute for Advanced Education and Research, Nagoya City University, Nagoya City Aichi Prefecture, Japan.

**Keywords:** Seaweed, Codium edule, Sulfated polysaccharide, Allergic contact dermatitis, Cytokines, Inflammation

## Abstract

***Purpose:*** Sulfated polysaccharide from *Codium* species has been reported for its antiinflammatoryactivities. However, the effect of sulfated polysaccharide from *Codium edule* on allergic responses has not been studied. The study was conducted to determine the effect ofsulfated polysaccharide (F1) from C. edule on allergic contact dermatitis (ACD) induced by2,4-dinitrofluorobenzene (DNFB) in female BALB/c mice.

***Methods:*** F1 was isolated using DEAE sepharose gel chromatography and chemically identifiedby LC-MS analyses. The effects of F1 on changes in ear thickness, allergic responses, andhistology were evaluated. The effects of F1 on the production of inflammatory cytokinesinterferon gamma (IFN-γ) and tumor necrosis factor-alpha (TNF-ɑ) in serum were also quantifiedand compared with standard prednisolone therapy.

***
Results:*** F1 was identified as a heteropolysaccharide with β-D-galactans and β-L-arabinans units.F1 was non-toxic at 2000 mg/kg. Administration of F1 in DNFB-challenged mice significantlysuppressed the increase in ear thickness, erythema, desquamation, and proliferation ofinflammatory cells. F1 significantly decreased the production of inflammatory markers, IFN-γand TNF-α in a dose-dependent manner when compared to the untreated group (*P* < 0.05).

***
Conclusion:*** Results suggest that F1 from *C. edule* is a bioactive sulfated heteropolysaccharidewith anti-inflammatory activity and might be a valuable candidate molecule for the treatmentof allergic diseases such as ACD.

## Introduction

Allergic contact dermatitis (ACD) is one of the commonest occupational skin diseases in the world. Being an acquired work-related disease, it has a great impact in terms of the socio-economic aspect of a country.^1^ ACD is usually characterized by the local inflammation of the skin triggered by the exposure to irritants or low-molecular-weight allergens known as haptens.^2^ ACD mainly involves two phases, namely sensitization and elicitation. The first reaction is the sensitization phase followed by the elicitation phase characterized by skin inflammation mediated by proinflammatory cytokines such as interferon gamma (IFN-γ) and tumor necrosis factor-alpha (TNF-ɑ).^3^ The disease is commonly treated with the use of glucocorticoids. However, the use of this class of drugs has an increased risk for undesirable effects such as hypertension, diabetes, and cataracts.

Seaweeds have been reported as potential sources of bioactive compounds.^4^ Marine algal sulfated polysaccharides possess numerous beneficial biological activities such as anticoagulant, antiviral, antitumor, antibacterial, anti-inflammatory, and antioxidant.^5-7^ Sulfated polysaccharides include fucoidan, ulvan, and carrageenan with chemical structures composed of sulfate groups attached to a polysaccharide backbone. The activities of these compounds are often associated with their molecular weight, sulfate content, and distinct molecular structure.^6,7^

*Codium edule*P.C. Silva, locally known as *Pocpoclo* or *pukpulo*, is a green seaweed seasonally available in the northern part of the Philippines. It is eaten raw as vegetable salad due to its high mineral content and palatable taste. It is used in traditional medicine as an insect repellent. In addition, *C. edule*is used in the treatment of wounds and inflamed skin due to its anthelmintic and antibacterial properties.^8^ However, studies on its bioactive compounds and metabolites are very limited. Despite the availability of commercial anti-inflammatory drugs in the Philippines, the use of herbal plants for the treatment of allergy and inflammation is still popular due to reports on the safety and efficacy of medicinal plants.^1^ However, there is a need to validate these reported folkloric uses with scientific evidence to assure that these plants are safe for use in local communities who cannot afford high-priced anti-inflammatory drugs.

The study was conducted to determine the efficacy of F1 from *C. edule* as a valuable anti-inflammatory molecule for the treatment of allergic diseases such as ACD. It aimed to characterize the polysaccharide (F1) from *C. edule* and determine its effects on allergic responses in ACD induced by 2,4-dinitrofluorobenzene (DNFB) in female BALB/c mice.

## Materials and Methods

### Algal materials

The fresh thalli of *C. edule* P.C. Silva were collected in Pagudpud, Ilocos Norte in June 2018. The alga was authenticated by Prof. Gavino C. Trono Jr., Ph.D. of the University of the Philippines Diliman Marine Science Institute (UP-MSI). A voucher specimen was likewise deposited at the UP–MSI (MSI No. 27907). The seaweeds were thoroughly washed with clean water to remove salt, epiphytes, and attached debris. The garbled algal sample was air-dried under shade for two weeks and ground using Wiley mill.

### Extraction of sulfated polysaccharide

The sulfated polysaccharide was extracted according to the method of Surayot et al with modifications.^9^ The milled sample was percolated in 95% ethanol in a 1: 10 ratio (w/v) overnight and decanted to obtain the residue. The residue was then extracted with distilled water (1:10 w/v) at 100°C for 4 hours. After cooling, the mixture was decanted and the supernatant liquid was collected and then added to 95% ethanol (1:1 v/v) to precipitate the crude polysaccharide (CP). CP was pelletized by centrifugation at 4°C and 1073 *× g* for two minutes. The pellet was then collected and deproteinized by Sevag method.^10^ Complete removal of protein from CP was confirmed by Bradford Assay.^11^ CP was purified by anion-exchange chromatography using DEAE-Sepharose Fast Flow Gel (Sigma DFF100-100ML). The CP solution (1 g/50 mL) was eluted using ultra-pure water and NaCl solutions (0.5 M to 2.0 M). All fractions were pooled together and labeled as “sulfated polysaccharide fraction” (F1). F1 was lyophilized and stored in an airtight amber bottle at -20°C until use.^12^

### Determination of chemical components of F1

The total sugar was determined by the phenol-sulfuric acid method using galactose as standard.^5^ The sulfate content was determined turbidimetrically by barium chloride-gelatin method using potassium sulfate as standard.^13^ The total uronic acid content was quantified colorimetrically by the sulfamate-dihydroxyphenyl method using galacturonic acid as standard.^14^ Concentration was identified regarding standard curves of the standards used.

### Fourier transform-infrared (FT-IR) spectroscopy

Two milligrams of F1 were pelletized with 200 mg of potassium bromide. The sample was scanned at a wavenumber range of 4000 to 400 cm^-1^ with a resolution of 4 cm^-1^ and 20 scans using Shimadzu IR Prestige 21. Peaks were analyzed and compared to the standard, Iota Carrageenan.^5^

### Liquid chromatography–mass spectroscopy** (**LC–MS) analysis

One hundred milligrams of F1 was hydrolyzed with 1.0 N H_2_SO_4_.^15^ The LC-MS analysis of the hydrolyzed sample was conducted using a Waters Xevo G2-XS QTOF mass spectrometer with an attached UPLC Waters CSH C18 column. A run time of 5.00 minutes was done using water + 0.1% Formic acid as solvent A (pH 2.7) and 95% Acetonitrile + 0.1% Formic acid as solvent B in a gradient manner. The sample was run at a capillary voltage of 3.0 kV, a cone voltage of 30 V, a collision energy of 6 V in a positive ion scan mode, and was positively ionized. A mass range of 50 to 3000 m/z using Leucine enkephalin (556.62 g/mol) as an internal reference standard and a scan time of 0.5 seconds was used.^16^

### Preparation of test animals

Six-week-old female BALB/c mice (20 to 25 g) were purchased from St. Luke’s Biotechnology and Research Division, Manila, Philippines. The animals were housed and acclimatized for seven days in standard plastic cages inside a controlled room environment with a room temperature of 18 to 25°C and a relative humidity of 60 ( ± 4) under a 12-hour dark/light cycle using artificial lighting. The animals were provided with unlimited access to food and water. All procedures were done according to the approved protocol by the University of Santo Tomas-Institutional Animal Care and Use Committee (UST-IACUC) and the Philippine Bureau of Animal Industry (Permit no. LAF - 017).

### Acute oral toxicity

Acute toxicity study was conducted following the guidelines of OECD 425. Five female BALB/c mice were dosed with 2000 mg/kg of F1 and were observed for 14 days for any signs of toxicity. All animals were sacrificed by cervical dislocation at the end of the experiment. Gross necropsy was done by a licensed veterinarian. The liver and kidneys were collected in buffered 10% formalin for histopathologic examinations.^17^

### DNFB-induced ACD and treatment in female BALB/c Mice

Thirty-six mice were randomly divided into six groups: Normal Control group DNFB (-), DNFB (+) induced group (Negative Control), 20 mg/kg Prednisolone group (Positive Control), 500 mg/kg F1group, 1000 mg/kg F1 group, and 2000 mg/kg F1 group. The shaved abdomen of each mouse (2 × 2 cm) was sensitized by painting 25 µL of 0.5% DNFB solution in an acetone-olive oil vehicle (4:1 v/v). All groups were challenged on the seventh day after sensitization by painting 5 µL of 0.1% DNFB solution to both sides of the right ear. The normal control was painted with the vehicle (acetone: olive oil) during sensitization and elicitation, while the DNFB-induced group was given a normal diet only after ACD induction. The positive control group received Prednisolone at a dose of 20 mg/kg BW. Experimental groups were administered with F1 (500 mg/kg, 1000 mg/kg and 2000 mg/kg BW) via oral gavage for seven days before elicitation. All mice were sacrificed on the eighth day and blood samples were collected via cardiac puncture. Serum levels of TNF-ɑ and IFN-γ were determined by enzyme-linked immunosorbent assay (ELISA).^1^

#### Measurement of ear swelling

The ear thickness was measured before elicitation (0 h) and 24 hours after DNFB challenge using a dial thickness gauge (Peacock Ozaki Japan, Model G-1A).^18^ Change in ear thickness in µm is expressed as PEM – BEM, where PEM and BEM represent post – elicitation and baseline measurements, respectively.

#### Evaluation of IFN-γ and TNF-α serum levels

The blood serum levels of IFNγ and TNF-α were measured by ELISA (BioLegend, USA) following the manufacturer’s protocol. The quantity in pg was calculated from standard curves of standard recombinant cytokines by a linear regression method. Percent inhibition was calculated using the equation (%) = (A_u_-A_t_)/A_t_ × 100, where A_u_ and A_t_ are the secreted cytokines (pg/mL) of the untreated group and treated groups, respectively.

#### Histopathologic examination of the challenged ear

The mice were sacrificed on the eighth day and the challenged ear was excised for histopathologic examination. The ears were fixed with 10% buffered formalin (4% formaldehyde, pH 6.9) and embedded in paraffin. The specimens were stained with hematoxylin and eosin (H & E) and evaluated by a licensed pathologist.^19^

### Statistical analysis

Results are presented as ± SEM of three measurements. One-way ANOVA and Tukey’s multiple comparison tests were used for statistical analysis using SPSS software. A *P* value of < 0.05 was considered statistically significant.

## Results and Discussion

### Chemical components of F1 and FT-IR spectroscopy

F1 afforded 19.12 ± 0.79%, 10.18 ± 0.53%, <1.0% of total carbohydrate, sulfate, and uronic acid contents, respectively. The carbohydrate content in F1 was comparatively lower than *C. corticum*that possessed 48.6%.^20^ The sulfate content of F1 was comparable with 8.97% observed in *C. intricatum*.^8^ The uronic content in F1 was lower than the reported 2% content in *C. fragile*.^9^

Sulfated polysaccharides present in marine algae are a class of compounds containing a carbohydrate backbone with a sulfate ester substitution in their sugar residues.^21^ The type and amount of sulfated polysaccharides present in algal cell walls are dependent on the season, environmental factors, and most especially, taxonomic grouping.^22^ The bioactivities of these macromolecules are attributed to their molecular weight, sulfate groups, and other anionic substituents.^23^ In green seaweeds, most polysaccharides extracted contain sulfates, rhamnose, xylose, and glucuronic acid except for *Codium*species which contain galactan as the major sugar backbone.^24-26^ The F1 extracted from *C. edule* P.C. Silva is generally made up of carbohydrate, sulfate, and a small amount of uronic acid.

**Figure 1:**
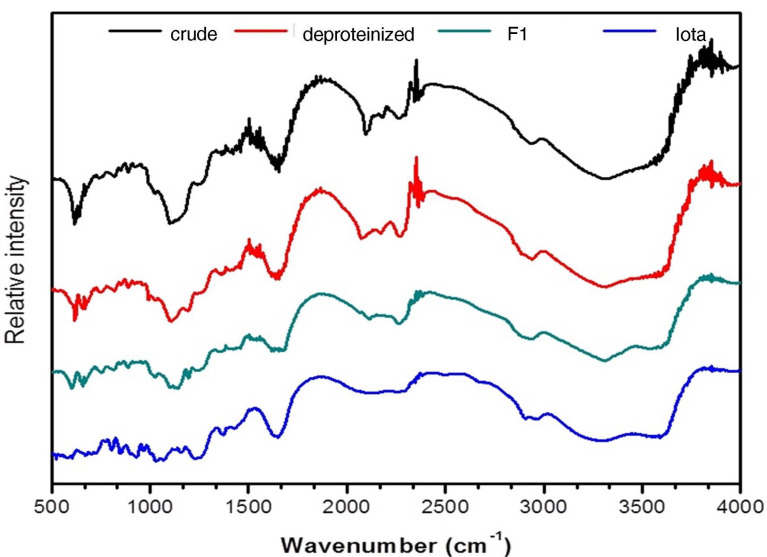
FTIR spectra of *Codium edule* F1 in comparison with crude, deproteinized and standard iota carrageenan.

The FT-IR spectra of F1 and iota carrageenan are presented in Figure 1. The presence of a broadband between 3500 and 3200 cm^-1^ signifies the presence of the stretching vibrations of the -OH groups present in polysaccharides.^26-28^ The bands between 1610 and 1648 cm^-1^ characterize the C = O stretching vibrations of the carboxylate group present in uronic acid, while the bands around 1418 cm^-1^ correspond to the carbonyl C-O stretching vibrations.^19,29^ The bands present between 1250 and 1370 cm^-1^ confirms the presence of the S = O stretching vibration of the sulfate groups present in polysaccharides. It is further described that the range between 1609 and 1420 cm^-1^ corresponds to the presence of uronic acid.^30^ Absorption bands observed within the signals of 1351 cm^-1^ and 1235 cm^-1^ correspond to S = O or sulfate ester groups that are distinctive components of sulfated polysaccharides. The bands found between 940 cm^-1^ and 930 cm^-1^ may be attributed to the C-O-C vibrations due to the presence of 3,6-anhydrogalactose and 3,6-anhydro-D-galactose residues, while the bands within the signals 815 to 830 cm^-1^ are a characteristic diagnostic region for sulfated galactans.^29,30^

Studies on the chemical composition of *C. edule* sulfated polysaccharide in terms of its functional groups are very limited. However, chemical and spectral data from the species *C. intricatum*and *C. divaricatum*were previously reported.^8,31^ The spectral data from the FTIR strongly suggest that F1 from *C. edule*contains sulfated galactans comparable with the sulfated polysaccharide extracted from *C. divaricatum*and *C. edule*.

### LC–MS analysis of F1 fragment

**Figure 2:**
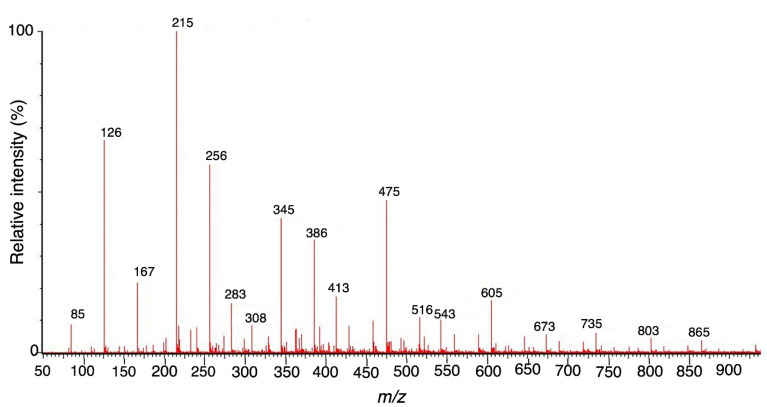
Full mass range scan (*m/z* 100-1000) of F1 fragment.

**Figure 3:**
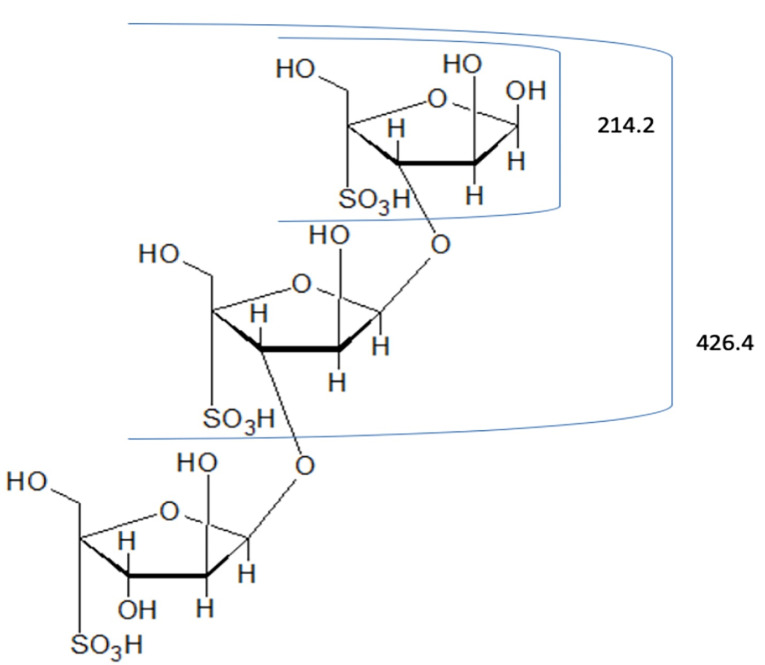
Proposed structure for the pentose fragment of F1. FI was assigned a putative identity of 3-linked β-arabinans with sulfate substitutions on C-2 or C-4.

**Figure 4:**
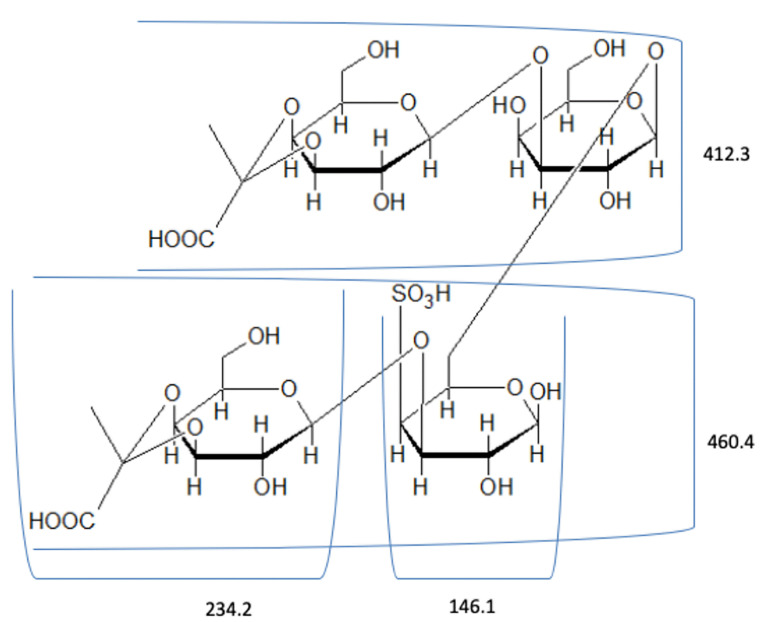
Proposed structure for the hexose fragment of F1. F1 was assigned a putative identity of a β--Gal*p*(3,4-Pyr)-(1→3)-β-Gal*p*-(1→6) linked to a β-Gal*p*(3,4-Pyr)-(1→3)-β- Gal*p*-(4SO3-).

The spectrum of hydrolyzed F1 obtained from LC–MS is shown in Figure 2. LC-MS Chromatograms of F1 showed polysaccharide linkage compositions that are usually seen in the chromatograms of different *Codium* species. Analyses revealed that F1 is a sulfated heteropolysaccharide with a backbone comprising pentose and hexose sugars. A putative identity for the sulfated pentose fragment observed mass around 654.6 m/z was a sulfated 3-linked β-arabinan with sulfate substitutions on C-2 or C-4 with a computed molecular weight of 654.6 + H and a molecular formula C_15_H_26_O_22_S_3_ (Figure 3). A sulfated hexose fragment observed at a mass around 412.3 m/z and 460.4 was assigned with a putative identity of a β-Gal*p*(3,4-Pyr)-(1→3)-β-Gal*p*-(1→6) linked to a β-Gal*p*(3,4-Pyr)-(1→3)-β-Gal*p*-(4SO_3_-), respectively. The sulfated hexose fragment has a computed molecular weight of 870.7 + H and a molecular formula of C_30_H_46_O_27_S as seen in Figure 4.

Thus, F1 was identified to be a sulfated heteropolysaccharide made up of a 3-linked β-D-galactans with ramifications on C-6 and terminal β-D-galactopyranose units with pyruvic acid ketal linked to *O*-3 and *O-*4 and a 3-linked pyranosic β-L-arabinans sulfated on C-2 and/or C-4.^32^

In a previous study, the components of the polysaccharide system present in *C. vermilara* were identified. The polysaccharide system consists of a fibrilar β-(1→4)–mannan, a β-(1 →3)–galactan which is highly sulfated and ramified, a β-(1→3)-arabinan which is linear and highly sulfated, and a linear β-(1→4) –mannan.^33^ A similar polysaccharide system was also observed in *C. decorticatum.*The major polysaccharide components of the species were a sulfated and pyruvylated 3- and 6--D-galactan, a sulfated 3-β-L-arabinan, and a 4-β-D-mannan with a low degree of sulfation.^20^ A pyruvylated sulfated galactan was also extracted from *C. divaricatum*that is mainly composed of (1→3)-β-D-galactopyranose residues.^31^ A highly pyruvylated sulfated galactan with linear backbone segments of 3-linked β-D-galactopyranose residues with a (1 →6) linkage was observed in *C. yezoense*. The sulfation was mainly concentrated on C-4 and C-6.^34^ The putative identities of the polysaccharide fragments from* C. edule*highly suggest that F1 is mainly composed of sulfated arabinans and galactans and is comparable to the polysaccharide system of other *Codium* species.

### Acute oral toxicity

**Figure 5:**
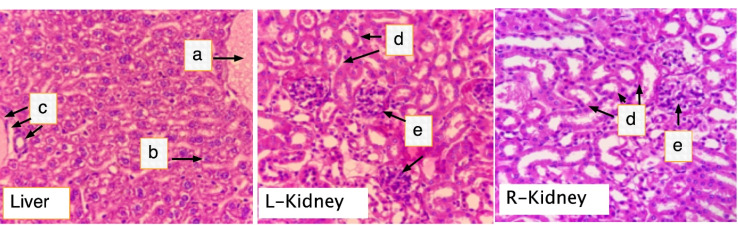
** Histological section of the liver and kidneys of rats treated with F1**. Mice were orally dosed with 2000 mg/kg BW FI for two weeks. On the 15^th^ day, all mice were killed, and liver and kidney samples were harvested for histolopathology analysis. (a) central vein; (b) Hepatocytes; (c) portal triad; (d) renal tubules; (e) glomerulus. (H & E, 400X).

The dose of 2000 mg/kg of F1 did not cause any signs of clinical toxicity to mice. All test animals were healthy and in good nutritional condition with no macroscopic parasites. The liver and kidney surfaces were all smooth with a firm consistency. Liver sections showed normal architecture without any sign of inflammation, necrosis, or fibrosis (Figure 5). The results of the toxicity assay revealed that the LD_50_ is beyond 2000 mg/kg. Polysaccharides from *Codium* species are generally non-toxic as observed in the polysaccharide fractions from *C. tomentosum and C. intricatum*which showed no toxic effects against normal human fibroblast cells.^8,35^

### Inhibitory effect of F1 on DNFB-induced ACD on female BALB/c mice

#### Ear swelling

**Figure 6:**
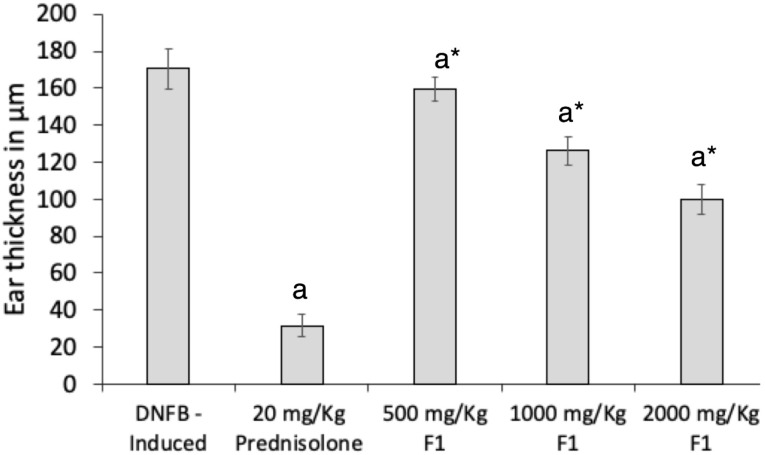
** Effect of F1 treatment on ear swelling of DNFB treated mice.** DNFB (-); DNFB + prednisolone; DNFB + F1 (500 mg/kg, 1000 mg/kg, 2000 mg/kg). Ear thicknesses were measured daily using a thickness gauge. Values are means ± SEM. of sixmice per group. ^a^ *P* < 0.05 vs. DNFB (-) mice, * *P* < 0.001 vs. Prednisolone.

Treatment with F1 significantly inhibited the increase in ear swelling in a dose-dependent manner in DFNB-treated mice (Figure 6). The average increase in ear thickness of groups dosed with 1000 mg/kg F1 and 2000 mg/kg F1 were 126.11 ± 6.3 µm and 100.00 ± 6.8 µm, respectively, and were significantly lower than the average ear thickness of the DNFB-induced group without treatment (*P* = 0.001, *P* = 0.002). As expected, treatment with prednisolone (20 mg/kg) exhibited the highest inhibition in ear thickness (*P* = 0.001).

Recently, a sulfated polysaccharide extracted from red algae was also shown to inhibit ACD responses. A porphyran at a dose of 2% aqueous solution effectively inhibited the ear swelling of mice in a TNCB-induced ACD model.^36^ A sacran, isolated from *Aphanothece sacrum*likewise inhibited the ear swelling in DNFB-exposed mice.^37^

ACD is an immunologic response against low-molecular-weight allergens known as haptens. Such a response is mediated by T-cells.^38^ In this study, ACD was induced on mice by using a chemical substance hapten known as DNFB. Haptens such as DNFB form a complex with skin proteins upon contact and function as immunogens. The immunogenic macromolecules are processed by antigen-presenting cells and presented to T-cells for activation.^39^ ACD, which is divided into sensitization and elicitation phases, was simulated in a seven-day induction and treatment experimental design. The most accurate and measurable physical attribute of ACD in a mice model is ear swelling. Ear swelling can be easily observed six hours post-elicitation and reaches its peak 24 hours post-elicitation and returns to normal after 48 to 72 hours.^40^

#### IFN-γ and TNF-α serum level inhibition

**Figure 7:**
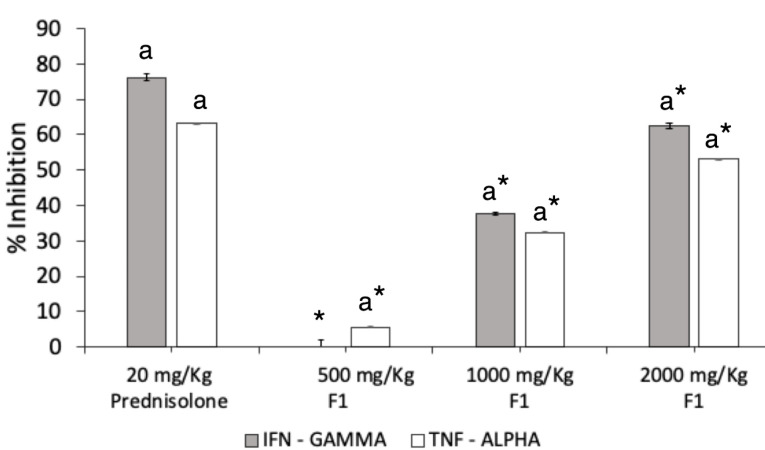
**
Effect of F1 treatment on IFN- **γ** and TNF-**α **production.** IFN-γ and TNF-α were quantified by ELISA in the serum obtained from DNFB-treated mice. F1 was orally given daily from the day of the first day of DNFB challenge (day 7). Levels of IFN- and TNF-α in the indicated groups were measured 24 h after the final administration. Values are means ± SEM of six mice per group. ^a^ *P* < 0.05 vs. DNFB (-) mice, * *P* < 0.001 vs Prednisolone.

DNFB challenge induced elevated production of IFN-γ and TNF-α levels in the serum of mice. A seven-day pre-treatment with FI significantly inhibited the production of both cytokines in ACD mice (Figure 7). The dose of 1000 mg/kg exhibited a percentage inhibition of 37.69 ± 0.2%, while a two-fold increase in percentage inhibition was observed at the dose of 2000 mg/kg at 62.42 ± 0.8%. The standard drug prednisolone displayed 76.24 ± 1.0% inhibitory activity which was significantly higher than all treatments with F1 (*P* = 0.001).

F1 at 2000 mg/kg displayed a notable TNF-α inhibition at 53.37 ± 0.06%, while 1000 mg/kg F1 exerted 33.19 ± 0.09% inhibitory effect. The dose of 500 mg/kg minimally inhibited the serum TNF-α level by 5.29 ± 0.12%. The inhibitory effect of F1 was still lower than the effect of prednisolone in regulating the production of the two inflammatory cytokines (*P* = .001).

Inflammatory response and pro-inflammatory cytokine upregulation play an important role in the pathophysiology of ACD and serve as a guide in targeting an adequate therapy for the disease.^41^ In a Th-1 directed reaction, the presence of TNF-α and IFN-γ is highly observed. In the case of ACD, the presence of these pro-inflammatory cytokines stimulates the proliferation of keratinocytes which results in skin hyperplasia or swelling. Furthermore, these two cytokines are also responsible for the upregulation of various chemokines leading to cellular infiltration in the inflamed area.^42^ The presence of TNF-α induces the production of other chemokines which results in the recruitment of leukocytes at the site of contact. Together with TNF-α, the presence of IFN-γ indicates a Th-1 skewing reaction. Thus, both TNF-α and IFN-γ play a significant role in the progression of ACD.^41^ Active skin cells further release TNF-α which causes increased immune cell infiltration in the epidermis. A reduction in TNF-α and IFN-γ levels may lead to decreased production of keratinocytes and inhibition of skin inflammation.^43^ A decrease in the levels of IFN-γ results in a downregulation of the Intracellular Adhesion Molecule 1 (ICAM-1) which prevents the infiltration of lymphocytes into the epidermis as well as the processing of antigen presentation. On the other hand, a decrease in the levels of TNF-α inhibits the maturation and migration of the Langerhans cells which are mainly responsible for inducing adaptive immunity after hapten exposure.^38^

It was revealed in the animal model that re-exposing the ear to DNFB of previously sensitized mice, induced ear swelling. Together with ear swelling, re-exposure to DNFB markedly increased the levels of the pro-inflammatory cytokines, TNF-α and IFN-γ. The changes brought about by re-exposure to DNFB to pre-sensitized mice mimics the human condition in an acute attack of ACD.^39^ The results revealed that the oral administration of *C. edule*F1 extract for seven days exhibited a significant suppressive effect on mice ear swelling compared to the untreated group.

#### Histopathologic analysis

**Figure 8:**
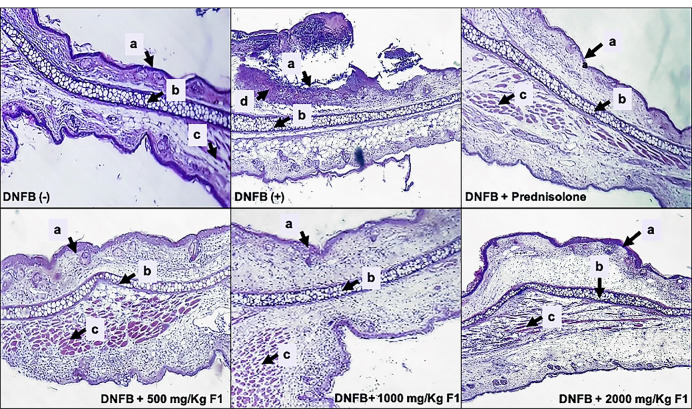
**
Histopathologies of the ear lesions in DNFB-treated mice after treatment with F1.** DNFB (-); DNFB (+), DNFB + prednisolone; DNFB + F1 (500 mg/kg, 1000 mg/kg, 2000 mg/kg). Ear sections were stained with hematoxylin and eosin (H & E, 100X). (a) stratified squamous epithelium, (b) cartilage, (c) muscle fibers, (d) inflammatory cells.

The effects of DNFB and F1 treatment on ear histology of mice were presented in Figure 8. The presence of a remarkable thickened keratinized stratified squamous epithelium with severe inflammation and skin ulceration was evidently seen as hallmarks of ACD.^41,42^ The ear tissues of mice treated with F1 showed normal cartilaginous tissue, few chronic inflammatory cells, and thickened keratinized stratified squamous epithelium with a moderate inflammation on the stroma similar to the histology of ear treated with the standard drug, prednisolone. The reduced inflammation in the elicited ears of the mice treated with F1 is attributed to the inhibition of the production of the pro-inflammatory cytokines TNF-α and IFN-γ seen in the serum 24 hours post-elicitation of the contact hypersensitivity response in mice. These cytokines are responsible for the activation of keratinocyte proliferation and cellular infiltration during the acute phase of ACD, and inhibition of their production by F1 resulted in the lowering of contact hypersensitivity responses in mice.

The current study demonstrated that sulfated plysaccharide (F1) from *C. edule* significantly suppressed the DNFB-induced skin inflammation and contact hypersensitivity responses, possibly by reducing the TNF-α and IFN-γ production from immune cells in mice. The efficacy of F1 from *C. edule*as a promising source of an agent that alleviates ACD symptoms should be given attention specifically for drug formulation. However, a drawback in utilizing marine polysaccharides is that they have low bioavailability due to their high molecular weights. Thus, it is very important to understand the structure-activity-relationship of marine polysaccharides to fully understand whether low-molecular-weight polysaccharides are more bioavailable than high-molecular-weight polysaccharides.

## Conclusion

In conclusion, F1 from *C. edule* significantly suppressed the DNFB-induced skin inflammation and contact hypersensitivity responses, possibly by reducing the TNF-α and IFN-γ production from immune cells in mice. These findings suggest that an anti-allergy component exists in sulfated polysaccharides of *C. edule*. The efficacy of F1 as a promising source of an agent that alleviates ACD symptoms should be given attention specifically for drug formulation. However, the data warrant further experiments on the isolation, elucidation of the specific chemical structure of F1, and more mechanistic assays in order to determine the bioavailability and exact mechanism of action as an anti-inflammatory agent in treating symptoms of ACD.

## Acknowledgments

The authors would like to acknowledge the assistance extended by Mr. John Paulin of the Department of Biochemistry, University of Santo Tomas, for determining the possible structure of FI.

The research is partially funded by the National Research Council of the Philippines (NRCP-02) and the Department of Science and Technology, Philippines (DOST).

## Ethical Issues

The study was conducted following the approved protocol of the University of Santo Tomas-Institutional Animal Care and Use Committee (UST-IACUC) and the Philippine Bureau of Animal Industry (Permit no. LAF - 017).

## Conflict of Interest

There are no conflicts to declare.
